# Diet Quality Among Hungarian Children Assessed Using the Healthy Eating Index-2020: Associations with Sociodemographic Factors

**DOI:** 10.3390/nu18142395

**Published:** 2026-07-22

**Authors:** Diána Sárga, Lajos Biró, Dániel Sándor Veres, Márta Veresné Bálint

**Affiliations:** 1 Health Sciences Division, Doctoral College, Semmelweis University, Üllői St. 26, 1085 Budapest, Hungary; veresne.balint.marta@semmelweis.hu; 2 Department of Dietetics and Nutritional Science, Faculty of Health Sciences, Semmelweis University, Vas St. 17, 1088 Budapest, Hungary; biro.lajos@semmelweis.hu; 3 Department of Biophysics and Radiation Biology, Semmelweis University, Tűzoltó St. 37-47, 1094 Budapest, Hungary; veres.daniel@semmelweis.hu

**Keywords:** children, diet quality, healthy eating index, sociodemographic factors

## Abstract

**Background**: Assessing overall diet quality has become increasingly important in nutritional epidemiology. The Healthy Eating Index (HEI) is one of the most widely used measures of diet quality; however, comparable data on Hungarian children are scarce. The current study aimed to assess the diet quality of Hungarian children using the Healthy Eating Index and to examine demographic factors associated with diet quality. **Methods**: This cross-sectional study included 666 children aged 4–10 years. Dietary intake was measured using three-day dietary records, and sociodemographic factors were collected via parental questionnaires. Diet quality was assessed using the Healthy Eating Index-2020 (HEI-2020). For the statistical analysis, linear regression, random forest models and one-way ANOVA with Tukey’s post hoc test were used. **Results**: The mean HEI score was 48.2 (SD 8.02), indicating low diet quality. Settlement type was significantly associated with the HEI score (*p* = 0.009). The multiplicity-corrected *p*-values for pairwise comparisons showed that children living in towns had significantly lower HEI scores (44.9, SD 7.97) than those living in county capitals (3.9, 95% CI:0.8–7.0, *p* = 0.006) and villages (−3.6, 95% CI: −0.61–−6.6, *p* = 0.011), but not significantly lower than those living in the capital (3.09, 95% CI: −0.25–6.4, *p* = 0.08). These differences were primarily related to whole-fruit and whole-grain component scores. Sex, age, and maternal education were not significantly associated with HEI score. The random forest model showed weak predictive performance (RMSE = 7.66). **Conclusions**: Diet quality among Hungarian children was generally suboptimal. The examined sociodemographic characteristics accounted for only a small proportion of the variability in the HEI score. This highlights the importance of ongoing research to understand dietary patterns and to uncover additional social, environmental, and behavioral aspects of dietary habits across cultures. Furthermore, the results indicate that interventions should also consider local food environments.

## 1. Introduction

Childhood overweight and obesity are growing epidemics and among the greatest challenges in the 21st century [1]. In 2022, more than 390 million children and adolescents lived with overweight or obesity worldwide [2]. In Hungary, according to the latest report of the European Childhood Obesity Surveillance Initiative (COSI), the prevalence of overweight and obesity is 23%, and the prevalence of underweight individuals is 13% [3,4]. Unfortunately, in many countries, children’s nutrient intake does not meet dietary recommendations [5].

Balanced nutrition and proper energy intake play a very important role in preventing noncommunicable diseases (NCDs), such as obesity. Furthermore, it is essential for children’s health, well-being and cognitive development. Childhood is a critical life stage for the development of dietary behaviours and preferences; therefore, it is important to establish healthy lifestyle habits during this period [6].

Dietary surveys play a crucial role in measuring dietary intake and identifying dietary patterns in targeted populations [5]. In recent decades, in nutrition epidemiology, measuring diet quality has become increasingly important, alongside nutrient intake analysis. Composite indices were developed to measure complex dietary patterns rather than individual factors, since people consume a combination of foods and nutrients. One of the most widely used indices of diet quality is the Healthy Eating Index (HEI), which has been extensively applied in surveillance, epidemiologic, and intervention research across the lifespan, starting at age 2. It has also been applied to many cultures and populations outside of the United States [7,8]. Despite its widespread international use, comprehensive assessments of the diet quality of Hungarian children using internationally comparable indices remain scarce [9].

The present study represents a secondary analysis of a previously established dataset [10]. Our previous publication described nutritional status, energy and nutrient intakes derived from three-day dietary records. The present analysis extends these findings by assessing overall diet quality using the HEI-2020, thereby complementing nutrient-based dietary assessment with a comprehensive measure of dietary patterns. To our knowledge, this is the first application of the Healthy Eating Index among Hungarian children. Furthermore, overall diet quality assessed, using the HEI-2020, and its associations with sociodemographic factors have not yet been investigated in this population. Therefore, the current study aimed to assess the diet quality of Hungarian children using the Healthy Eating Index and to examine demographic factors associated with diet quality.

## 2. Materials and Methods

### 2.1. Study Design, Sample Size, and Sampling

In this cross-sectional study, 733 4–10-year-old Hungarian children were initially included; the final sample consisted of 666 individuals with valid dietary records, including 252 aged 4–6 years and 414 aged 7–10 years. The gender distribution was 49.4% female (*n* = 329) and 50.6% male (*n* = 337). Participants were recruited using a combination of in-person snowball sampling and telephone and online contact. In smaller settlements, recruitment was initiated through schools and the health visitor network, after which participating families referred to additional eligible families. In larger settlements, participants were recruited through a qualitative recruitment network using telephone and online contact. Children were recruited from different regions and settlements across the country. Quota sampling was applied, where, after weighting, the population proportions were approximated by age group, sex, settlement size, and region. In the present study, all analyses were conducted on the unweighted sample (*N* = 666).

### 2.2. Assessment of Anthropometric Data and Sociodemographic Factors

Anthropometric measurements were performed by trained dietitians according to the National Longitudinal Study of Child Growth protocol [11]. Body weight and height were assessed using calibrated digital scales and portable stadiometers. Body mass index (BMI) was defined, and nutritional status was classified as ‘underweight’, ‘normal weight’, ‘overweight’ or ‘obese’ on the basis of sex- and age-specific cut-off points (2*z-score thresholds) as outlined by Cole et al. [12,13,14]. Sociodemographic factors, including household income, maternal education, geographical region, and type of settlement, were collected via a structured parental questionnaire. To ensure consistency and data completeness, the questionnaires were verified by trained field researchers onsite at the same time as the anthropometric measurements were taken.

### 2.3. Assessment of Dietary Intake

Dietary intake was measured using a three-day dietary record completed by parents and caregivers and validated by trained dietitians selected by the Hungarian Dietetic Association. Nutrient intake data were recorded and calculated via NutriComp DietCAD 5.0 software (NutriComp Ltd., Budapest, Hungary) [15]. Invalid dietary records were identified using the Goldberg method and were excluded from further analysis due to the high probability of under- or overreporting of dietary intake, resulting in the final sample size [16].

### 2.4. Healthy Eating Index-2020 (HEI-2020)

The diet quality of the participants was assessed via the Healthy Eating Index-2020 (HEI-2020), based on publicly available methods, guidelines and scoring criteria [8,17,18]. The index consists of 13 components, divided into two subgroups: adequacy and moderation. The food groups within the adequacy components that reflect a healthy dietary pattern are total fruits, whole fruits, total vegetables, greens and beans, whole grains, dairy, total protein foods, seafood and plant proteins, and fatty acids. The other group comprises the moderation components that are recommended to be limited in the everyday diet, such as refined grains, sodium, added sugars, and saturated fats. Higher scores within the adequacy components indicate higher, more desirable consumption, and higher scores within the moderation components indicate lower consumption. The intake between the minimum and maximum scores is calculated in proportion to the difference between them. Summing the scores of the 13 components yields a final HEI score ranging from 0 to 100 [8]. A higher score indicates better adherence to the dietary guidelines and better diet quality [19]. To enable HEI-2020 calculation, all food items and meals recorded in the dietary survey data were disaggregated to ingredients. A total of 825 unique items were identified and paired with the corresponding items in the Food Patterns Equivalents Database (FPED) and Food Patterns Equivalents Ingredients Database (FPID) [17]. As several local foods and ingredients lacked direct equivalents in the database, nutrition expert judgment was used to identify the most relevant matches based on composition and culinary use. The resulting database enabled us to convert gram-based intake to cup equivalents, as required for HEI score calculations. The FPED and FPID provided the Food Pattern conversion factors expressed as cup or ounce equivalents per 100 g of food. Individual Food Pattern Equivalents were derived by applying these conversion factors to the reported food amounts, averaging intakes across the three dietary assessment days, and standardizing the results per 1000 kcal of energy intake [17]. Exceptions were the Saturated Fats and Added Sugars components, which were calculated as a percentage of total energy intake. Subsequently, the 13 HEI-2020 dietary components were derived according to the HEI-2020 methodology. Component scores were then calculated using the HEI-2020 scoring standards and summed to obtain the total HEI-2020 score for each participant [8,20].

### 2.5. Statistical Analysis

We applied a linear regression model to quantify the effects of sociodemographic factors (maternal education, income, region, settlement, sex, and age) and BMI on the total HEI score. In the final linear regression model, we analyzed maternal education, age, sex, settlement and BMI to measure their effects on HEI. As 40% of the respondents did not know or did not wish to provide their household income level, this variable had to be excluded from the model. Because the region and settlement type were multicollinear, they had to be treated in separate models, and the final model examined included the settlement type. To assess potential two-way interactions, we examined descriptive plots, calculated information criteria (Akaike Information Criterion [AIC] and Bayesian Information Criterion [BIC]), and compared models with and without specific interactions via likelihood ratio tests (LRTs). Since the LRTs, AIC, and BIC did not indicate improvement with interactions, and the descriptive plots did not reveal any meaningful interactions, no interactions were included in the final models. Diagnostic plots on the final model indicated no significant issues with multicollinearity, linearity, homoscedasticity, or normality. When an overall association was observed for a categorical variable (*p* < 0.10), multiplicity-corrected *p*-values were calculated to allow pairwise comparisons of their levels. For predictive purposes, to determine whether the demographic factors could predict the HEI score, we applied a machine learning approach using random forest models. The dataset was split into a 60:40 train–test ratio. Random forest models with 500 trees were fitted, and hyperparameters (number of variables considered at each split and minimum node size) were optimized via 10-fold cross-validation by minimizing the mean squared error (MSE), via the Ranger engine and permutation-based variable importance. These statistical analyses were created by using R software (v4.5.1) (R Foundation for Statistical Computing, Vienna, Austria) [21]. To identify which dietary components contributed to the observed differences in overall HEI score across settlement types, differences in HEI component scores were assessed using a one-way ANOVA with Tukey post hoc tests in IBM SPSS Statistics for Windows, Version 30.0 (IBM Corp., Armonk, NY, USA).

## 3. Results

### 3.1. Sociodemographic Characteristics

Sociodemographic characteristics, including maternal education, region, type of settlement, and household income, are presented in Table 1. The mean age of the participants was 7.31 years (SD 1.99). The proportions of females (49.4%) and males (50.6%) were approximately equal. The mean BMI was 17.0 kg/m^2^ (SD 3.25 kg/m^2^), and 60.7% of the children were classified as normal weight. The mean value of the Healthy Eating Index (HEI) score was 48.2 (SD 8.02), with a median of 48.2 (Q1:42.5; Q3: 53.7), and a range from 28.4–71.1.

### 3.2. Healthy Eating Index-2020 Associations with Sociodemographic Factors

The results of the linear regression examining the associations between the HEI score and maternal education, type of settlement, BMI, sex, and age are presented in Table 2. Maternal education was not significantly associated with the HEI score (*p* = 0.13), and the magnitude of this effect was small (partial R^2^ = 0.007). Compared to primary/vocational education, neither secondary (−1.55, 95% CI: −0.09–3.19, *p* = 0.064) nor tertiary education (1.52, 95% CI: −0.14–3.17, *p* = 0.072) was associated with HEI score. BMI (−0.10, 95% CI: −0.30 to 0.09, *p* = 0.30), sex (−1.17, 95% CI: −2.41–0.07, *p* = 0.064) and age (−0.09, 95% CI: −0.41–0.24, *p* = 0.6) did not have a significant association with HEI score. Although HEI score is not presented in Table 2. male participants (47.8, SD 8.26) had slightly lower HEI scores than females (48.8, SD 7.75) did, and the HEI decreased marginally with increasing age and BMI.

Among the variables examined, settlement type was the only one significantly associated with the HEI score (*p* = 0.009), although the magnitude of this effect was small (partial R^2^ = 0.018).

To further explore this association, the results of the multiplicity-corrected *p*-values for pairwise comparisons are presented in Table 3. This comparison revealed that children living in towns had significantly lower HEI scores than those living in county capitals (3.9, 95% CI 0.8–7.0, *p* = 0.006) and villages (−3.6, 95% CI −6.6–−0.61, *p* = 0.011), but not significantly lower than those living in the capital (3.09, 95% CI −0.25–6.4, *p* = 0.08).

### 3.3. Overall HEI and Component Scores by Settlement Type

The total HEI score and HEI component scores for the overall sample and by settlement type, together with the maximum achievable score for each component are presented in Table 4. Across the total sample, the adequacy components of greens and beans, whole grains, seafood and plant proteins received low scores. Among the moderation components, participants achieved relatively high scores for added sugars, but sodium showed an extremely low mean score. Post hoc analyses revealed that children living in towns had significantly lower component scores for whole fruit and whole grains than did those living in all other settlement types. With respect to total fruit intake, children living in towns had lower scores than those living in the capital city and county capitals, whereas no significant difference was observed between children living in towns and villages.

**Figure 1:**
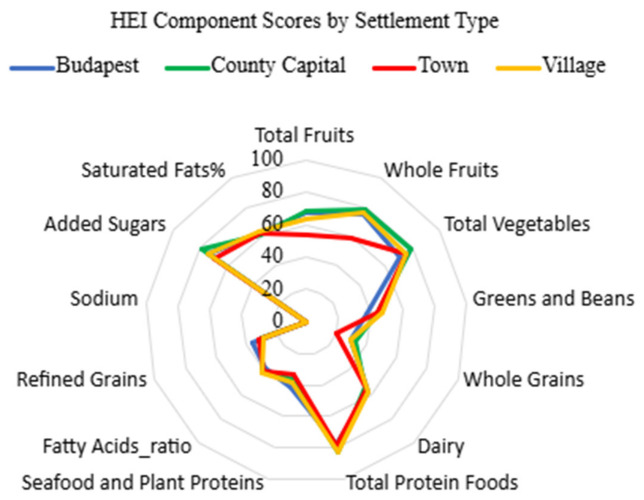
Radar plot of the mean Healthy Eating Index component scores by settlement type. On the radar plot, the 13 component scores are shown as percentages of their maximum values, where the outer edge represents 100% of the maximum score, while the centre represents 0% of the maximum score for each component [8]. The colored lines represent the mean scores for children living in Budapest (blue), county capitals (green), towns (red), and villages (yellow). Higher values reflect greater adherence to the dietary recommendations for the corresponding dietary component.

The differences in HEI component scores by settlement type are visualized in Figure 1.

### 3.4. Random Forest Analysis

**Figure 2:**
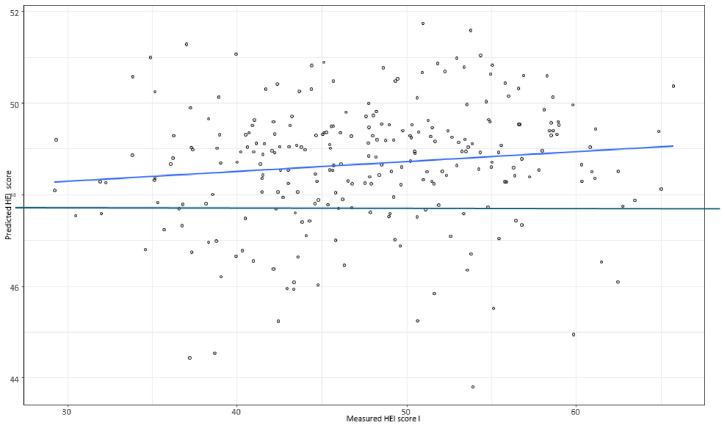
Random forest model: measured and predicted HEI scores. The scatter plot in Figure 2 shows the relation between measured and random forest-predicted Healthy Eating Index (HEI) scores. Each point represents one participant. The smooth blue line represents the fitted linear regression line illustrating an assumed linear relation between measured and predicted HEI scores, while the horizontal blue line represents the mean predicted HEI score.

Although the random forest analysis identified BMI, maternal education, and settlement type as the most important predictors of the HEI score, the model demonstrated weak predictive performance (RMSE = 7.66). The predicted values showed low variability, and the relationship with the observed HEI scores also remained low, as shown in Figure 2.

## 4. Discussion

The current study was conducted to assess diet quality using the Healthy Eating Index among Hungarian 4-to-10-year-old children. It builds on our previous analysis of the same cohort, which identified several nutritional inadequacies, including low consumption of fruits, vegetables, and whole grains, excessive sodium intake, and added sugar intake exceeding the WHO recommendation [10].

In the present study the overall low mean HEI score 48.2 (SD 8.02), and the unfavorable component scores suggest a generally suboptimal diet quality. The component scores for the total sample were especially low for greens and beans, whole grains, seafood and plant proteins, which are essential parts of balanced nutrition. These findings are consistent with our previous nutrient intake analysis of the same study population, in which only 43% of the children consumed whole-grain products at least once during the three-day dietary assessment, while the mean fruit and vegetable intake was only 263 g/day, well below the recommended 400 g/day [10]. A near-zero score for sodium indicates that sodium intake was substantially above the recommended level in the examined population. The mean daily sodium intake was 3362 mg/day, as reported in our previous analysis of the same study population, based on three-day dietary records [10]. Added sugar received one of the highest component scores among the moderation components, at 7.47 (SD 2.45). Although the relatively higher HEI component score for added sugar indicates better adherence to dietary recommendations, our previous analysis showed that added sugars accounted for approximately 11% of total energy intake, exceeding the WHO recommendation (<10% of total daily energy intake) [6,10].

Among studies using the Healthy Eating Index, it was observed that children’s diet quality varies by country but typically remains low. For example, the median HEI score among Japanese preschoolers was reported to be 50 for females and 51 for males [22]. In a Greek study from 2016, scores of 65.17 for boys and scores of 66.47 for girls were reported [23]. Recent European studies using other validated diet quality indices have similarly reported poor adherence to dietary recommendations. A Portuguese study using the Diet Quality Index-International (DQI-I) reported generally low diet quality, characterized by inadequate vegetable, fruit, grain and fibre intake together with excessive sodium intake [24]. Likewise, a recent Croatian national dietary survey, using the Diet Quality Index for Adolescents (DQI-A), found moderate overall diet quality and inadequate adherence to dietary recommendations [25]. Furthermore, the multicountry DELICIOUS Project, using the Youth Healthy Eating Index (YHEI), demonstrated suboptimal adherence to dietary recommendations among children and adolescents across five Mediterranean countries [26].

In our study, most sociodemographic variables, sex, age and maternal education, were not significantly associated with HEI scores. Girls’ HEI score 48.8 (SD 7.75) was slightly higher than boys’ 47.8 (SD 8.26), but the difference was negligible. The HEI score marginally decreased with increasing age, and children of mothers with higher education had a slightly higher HEI score than those whose mothers had primary or vocational education, but these differences did not reach statistical significance. Several previous studies have described associations with diet quality, parental education, gender, age, and income. Studies conducted among Spanish and Swedish adolescents revealed that diet quality, among other factors, was associated with gender (girls had better diet quality than boys did) and with parents’ educational level. Higher education is especially associated with higher intake of fruit and vegetables [27,28]. Similar results were reported in a Portuguese study, in which parental education and income were associated with increased fruit and vegetable consumption [29]. According to the National Health and Nutrition Examination Survey (NHANES), dietary quality in the United States does not meet the dietary guidelines. The average HEI score between 2 and 58 years is 58, and the HEI score by age group was recorded as follows: 2–4 years = 58.3; 5–8 years = 52.6; 9–13 years = 50.1; this also shows a decreasing trend, as in the current research [8]. Another study examining dietary and nutritional intake behaviors among early adolescents (10–13 years old) in the United States revealed that males, adolescents from poorer households, and adolescents with lower parental education had lower diet quality [30]. An Iranian study reported that younger children (6–12 years old) had higher diet quality than did adolescents (13–18 years old) and that individuals from families with moderate or high socioeconomic status had higher diet quality [31].

In our study, the only statistically significant association was between the HEI score and settlement type, where those living in towns had significantly lower HEI scores than those living in settlements. In the statistical analyses, the effect size was small, with a few-point difference, suggesting limited practical applicability. Some studies discuss the association between diet quality and place of living. One study found conflicting results across the literature. In a Finnish study, children living in semiurban areas had lower diet quality than those living in urban areas; meanwhile, in South-West Britain, the opposite was observed [28]. An Australian study revealed that fruit consumption was higher in smaller rural towns than in regional centers [32]. Similarly, in our research, a post hoc analysis suggested that children living in villages had whole-fruit HEI component scores very similar to those of children living in the capital city and county capitals, whereas children living in towns had significantly lower scores. Another significant difference in scores was observed for whole-grain components. The reasons for these disparities remain unclear, as these factors were not examined in this research and would require further research. However, differences in food availability, food purchasing patterns, nutrition knowledge, the food environment, and sociological factors may explain these variations. Importantly, Hungarian research on nutrient intake among children and adults generally reports lower consumption of whole grains, fruits, and vegetables than the dietary recommendations [4,33,34,35].

These results highlight the importance of ongoing research to better understand dietary patterns and to uncover additional social, environmental, and behavioural aspects of dietary habits across cultures, influencing children’s diets. They also suggest that future nutrition interventions should also consider local food environments. The present study provides the first assessment of diet quality using the HEI-2020 among Hungarian children, offering a more comprehensive evaluation of dietary patterns than nutrient-based analyses alone. In addition, it establishes a methodological framework for future HEI-based studies in other Hungarian populations and facilitates international comparisons using a standardized diet quality metric. These findings may contribute to the development of evidence-based nutrition policies and targeted public health interventions.

The strengths of this study are that, to our knowledge, no HEI analyses have been conducted among children in Hungary, and no associations with HEI scores have been examined in this age group. HEI analysis provided us with a more nuanced understanding of the dietary patterns of Hungarian children, complementing nutrient intake research. The disaggregated food database used for this research enables us to conduct further HEI analysis in Hungary across different age groups, using a robust methodology. The limitations of this study are that, owing to the low response rate regarding income, we had to exclude this variable from our model, which could have been an asset for analysing economic factors in our research. Furthermore, the cross-sectional study design does not allow for the measurement of long-term effects, for example, with age.

## 5. Conclusions

Diet quality among Hungarian 4–10-year-old children is generally suboptimal, as reflected in the low overall mean HEI score and unfavorable component scores. Among the examined variables, settlement type had a significant association with HEI score. Children living in towns had significantly lower HEI scores than those living in county capitals and villages, but not significantly lower than those living in the capital. We found no significant association between sex, maternal education, BMI or age and diet quality. However, females had slightly higher HEI scores than males. The HEI scores were modestly decreasing with age and BMI and slightly increasing with maternal education. In conclusion, sociodemographic factors had limited explanatory power for diet quality among Hungarian children aged 4–10 years. This finding was further supported by the random forest model, which showed poor predictive performance for the Healthy Eating Index, when sociodemographic variables were used. These findings suggest that diet quality may be influenced by several factors beyond sociodemographic characteristics.

## Figures and Tables

**Table 1:** Demographic characteristics of the study participants.

Variable	Overall (*N* = 666)
**Age** (**years**)	7.31 (1.99)
**Sex**	
Female	329 (49.4%)
Male	337 (50.6%)
**Maternal education**	
Primary/Vocational	158 (23.7%)
Secondary	230 (34.5%)
Tertiary	252 (37.8%)
Missing	26 (3.9%)
**Region**	
Central	178 (26.7%)
East	308 (46.2%)
West	180 (27.0%)
**Settlement**	
Capital city	129 (19.4%)
County capital	214 (32.1%)
Other town	60 (9.0%)
Village	263 (39.5%)
**Household income** (**1000 HUF**)	338 (125)
Missing	268 (40.2%)
**BMI** (**kg/m^2^**)	17.0 (3.25)
**BMI category**	
Underweight	92 (13.8%)
Normal weight	404 (60.7%)
Overweight	110 (16.5%)
Obese	60 (9.0%)
**HEI score**	48.2 (8.02)

Values are presented as mean (SD) or *n* (%).

**Table 2:** Results of the linear regression analysis of factors associated with HEI score.

Characteristic	*p*	Partial R^2^	β (95% CI)	Std β (95% CI)
**Education of the Mother**	0.13	0.007		
Primary and Vocational				
Secondary	0.064		1.55 (−0.09–3.19)	0.09 (−0.01–0.19)
Tertiary	0.072		1.52 (−0.14–3.17)	0.09 (−0.01–0.19)
**Settlement**	0.009	0.018		
Capital City				
County Capital	0.4		0.81 (−1.01–2.63)	0.05 (−0.06–0.15)
Other Town	0.017		−3.09 (−5.63–−0.55)	−0.11 (−0.20–−0.02)
Village	0.6		0.50 (−1.30–2.30)	0.03 (−0.08–0.14)
**BMI [kg/m^2^]**	0.3	0.002	−0.10 (−0.30–0.09)	
**Sex**	0.064	0.005		
Female				
Male	0.064		−1.17 (−2.41–0.07)	−0.07 (−0.15–0.00)
**Age [years]**	0.6	0	−0.09 (−0.41–0.24)	

Abbreviation: CI = Confidence Interval. Primary and Vocational Education, Capital City, and Female were used as reference categories in the regression model.

**Table 3:** Pairwise comparisons of mean HEI scores by settlement type.

Group Comparison	Difference	95% CI	*p*-Value
Budapest–County capital	−0.808	−3.197–1.581	0.8196
Budapest–Town	3.089	−0.245–6.423	0.0807
Budapest–Village	−0.500	−2.858–1.859	0.9477
County capital–Town	3.897	0.842–6.952	0.0059
County capital–Village	0.309	−1.642–2.26	0.9771
Other town–Village	−3.588	−6.566–−0.61	0.0107

Abbreviation: CI = Confidence Interval. Pairwise comparisons were performed using Tukey’s adjustment for multiple comparisons.

**Table 4:** Healthy Eating Index component scores by total sample and by settlement (mean (SD)).

HEI Components	Maximum Score	Total (*N* = 666) Mean (SD)	Capital City (*n* = 129) (SD)	County Capital (*n* = 214) (SD)	Town (*n* = 60) (SD)	Village (*n* = 263) (SD)	*p*-Value (ANOVA)
**Total HEI score**		48.2 (8.02)	48.1 (8.76)	49.0 (7.57)	44.9 (7.97)	48.5 (7.87)	
**Adequacy components**							
Total Fruits	5	3.25 (1.61)	3.38 (1.54) ^b^	3.44 (1.60) ^b^	2.67 (1.76) ^a^	3.15 (1.60) ^ab^	**0.005**
Whole Fruits	5	3.77 (1.69)	3.77 (1.69) ^b^	3.93 (1.61) ^b^	2.93 (1.95) ^a^	3.83 (1.65) ^b^	**<0.001**
Total Vegetables	5	3.75 (1.21)	3.52 (1.33) ^a^	3.93 (1.11) ^b^	3.72 (1.20) ^ab^	3.73 (1.22) ^ab^	**0.023**
Greens and Beans	5	2.20 (1.74)	1.90 (1.71)	2.19 (1.79)	2.24 (1.77)	2.35 (1.70)	0.115
Whole Grains	10	2.96 (2.39)	2.97 (2.49) ^b^	3.23 (2.41) ^b^	1.97 (1.79) ^a^	2.96 (2.41) ^b^	**0.004**
Dairy	10	5.61 (2.66)	5.79 (2.64)	5.39 (2.60)	5.55 (2.98)	5.71 (2.65)	0.483
Total Protein Foods	5	4.08 (0.99)	3.87 (1.08)	4.14 (0.92)	3.96 (1.08)	4.16 (0.97)	**0.024**
Seafood and Plant Proteins	5	1.89 (1.80)	2.06 (1.75)	1.84 (1.79)	1.66 (1.73)	1.90 (1.85)	0.521
Fatty Acids	10	4.06 (2.56)	3.79 (2.54)	4.07 (2.53)	4.03 (2.67)	4.20 (2.56)	0.516
**Moderation components**							
Refined Grains	10	3.04 (2.87)	3.54 (3.21)	2.92 (2.70)	3.15 (3.00)	2.87 (2.78)	0.150
Sodium	10	0.02 (0.20)	0.03 (4.05)	0.00 (0.00)	0.00 (0.00)	0.03 (7.16)	0.329
Added Sugars	10	7.47 (2.45)	7.38 (2.53) ^ab^	7.85 (2.06) ^b^	6.86 (3.00) ^a^	7.34 (2.53) ^ab^	**0.021**
Saturated Fats	10	6.16 (3.01)	6.16 (3.05)	6.06 (2.91)	6.18 (2.73)	6.23 (3.14)	0.944

HEI component scores are presented as mean (standard deviation). Differences between groups (Budapest, county capitals, towns, and villages) were assessed using one-way ANOVA, and post hoc pairwise comparisons were performed using Tukey’s HSD test. Bold *p*-values indicate statistically significant differences according to the ANOVA. Values sharing at least one common superscript letter are not significantly different according to post hoc test (*p* < 0.05). ANOVA indicated a statistically significant difference in total protein food scores across settlement types, but the post hoc comparisons test revealed no consistent pairwise differences between groups.

## Data Availability

The data presented in this study are available on request from the corresponding author due to ethical and privacy restrictions. The study involved minors and public sharing of individual-level data was not included in the informed consent procedure.

## Abbreviations

The following abbreviations are used in this manuscript:

AIC	Akaike Information Criterion
BIC	Bayesian Information Criterion
BMI	Body Mass Index
CI	Confidence Interval
COSI	European Childhood Obesity Surveillance Initiative
HEI	Healthy Eating Index
FPED	Food Patterns Equivalents Database
FPID	Food Patterns Equivalents Ingredients Database
LRT	Likelihood Ratio Tests
NCDs	Noncommunicable Diseases
NHNES	National Health and Nutrition Examination Survey
SD	Standard Deviation
WHO	World Health Organization
